# Atypical presentation of giant fibroadenoma of the breast

**DOI:** 10.11604/pamj.2021.39.168.26975

**Published:** 2021-07-05

**Authors:** Ismail Abdul Sattar Burud, Norfarizan Azmi

**Affiliations:** 1 Department of Surgery, International Medical University, Clinical Campus, Seremban, Malaysia,; 2 Department of Surgery, Hospital Tuanku Ja´afar, Seremban, Malaysia

**Keywords:** Fibroadenoma, breast, woman

## Image in medicine

A 36-year-old multiparous woman presented with a slow-growing left breast lump which was increasing in size over the past five years. It was 5x5 cm when she first noticed it. It did not bother her and hence she did not seek medical advice. Initially, there was no pain but for the past six months, she complained of dull aching pain and discharge from the lump. She had no family history of cancer. On examination the right breast was normal. There was a cauliflower-like ulcerative growth of 30x30 cm occupying the whole of the left breast (A). The surface was nodular and there was serosanguinous discharge from the area of ulceration. The lump was firm to hard in consistency and freely mobile with no fixity to the underlying muscle. Small axillary lymph nodes were palpable. Computerized tomography showed the lobulated lump of heterogenous density predominantly hyperdense with areas of hypodensity occupying the whole of the left breast (B). Wedge biopsy showed proliferation of epithelial and stromal cells suggestive of fibroadenoma with no malignant cells. She underwent mastectomy with primary closure and skin grafting. The weight of the breast lump was 2.6 kg. Histopathologic examination showed stroma consisting of proliferating ducts in slit-like pattern and pericanalicular areas. The stroma was fibromyxoid in nature and minimally cellular with no malignancy. Features were consistent with giant fibroadenoma.

**Figure 1:**
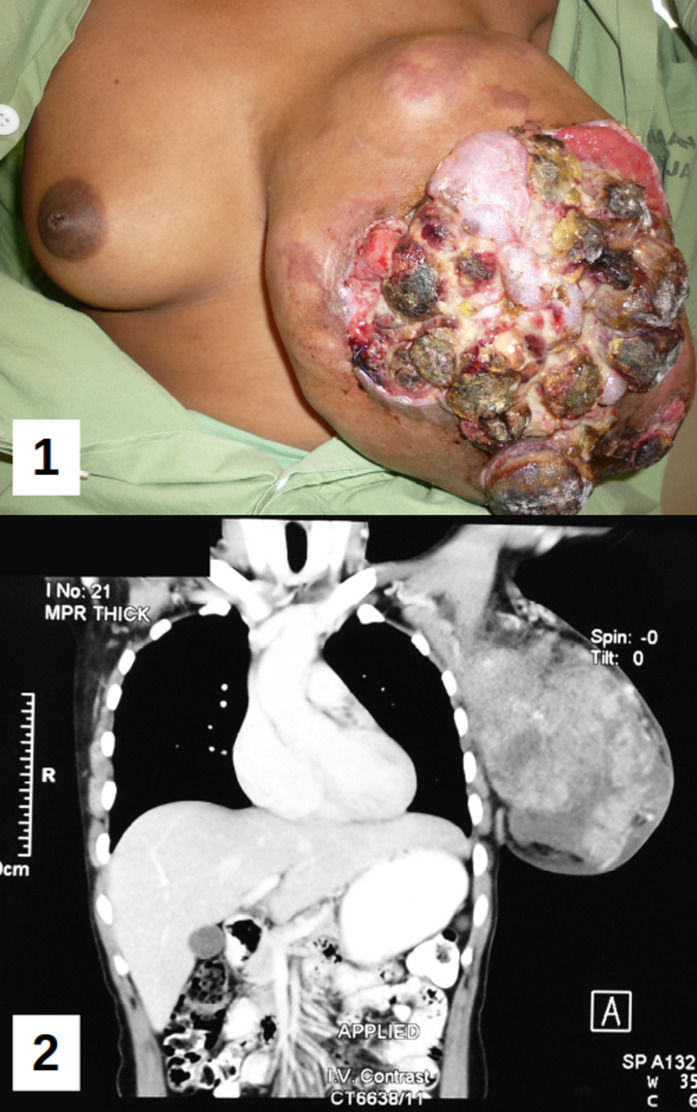
A) ulcerative cauliflower like Lump of the left breast; B) computed tomography (CT) image of the left breast showing hypo and hyperdense areas

